# Neural mechanisms to predict subjective level of fatigue in the future: a magnetoencephalography study

**DOI:** 10.1038/srep25097

**Published:** 2016-04-26

**Authors:** Akira Ishii, Masaaki Tanaka, Yasuyoshi Watanabe

**Affiliations:** 1 Department of Physiology, Osaka City University Graduate School of Medicine, 1-4-3 Asahimachi, Abeno-ku, Osaka 545-8585, Japan; 2 RIKEN, Center for Life Science Technologies, 6-7-3 Minatojima-minamimachi, Chuo-ku, Hyogo 650-0047, Japan

## Abstract

Fatigue is a major contributor to workplace accidents, morbidity, and mortality. To prevent the disruption of homeostasis and to concurrently accomplish an assigned workload, it is essential to control the level of workload based on the subjective estimation of the level of fatigue that will be experienced in the near future. In this study, we aimed to clarify the neural mechanisms related to predicting subjective levels of fatigue that would be experienced 60 min later, using magnetoencephalography. Sixteen healthy male volunteers participated in this study. In relation to the prediction, a decrease of alpha band power in the right Brodmann’s area (BA) 40 and BA 9 at 1200 to 1350 ms and that in the right BA 9 at 1350 to 1500 ms, and a decrease of gamma band power in the right BA 10 at 1500 to 1650 ms were observed. In addition, the decreased level of alpha band power in BA 9 at 1200 to 1350 ms was positively associated with the daily level of fatigue. These findings may help increase our understanding of the neural mechanisms activated to indicate the need to take a rest based on the prediction of the subjective fatigue in the future.

Fatigue is prevalent in modern societies. It has been reported that 20–30% of the general population in Europe and the United States experience substantial fatigue^1^,^2^,^3^,^4^,^5^, and in Japan, more than half of the adult population reports experiencing fatigue^6^. In addition, fatigue is a major contributor to workplace accidents, morbidity, and mortality^7^,^8^. Thus, it is important to clarify the mechanisms of fatigue and, if possible, to overcome fatigue based on these mechanisms.

Fatigue is defined as a decline in the ability to perform or the efficiency of performance of mental and/or physical activities caused by excessive mental or physical activity or disease and is often accompanied by a peculiar sense of discomfort, a desire to rest, and a decline in motivation, referred to as fatigue sensation^9^. Fatigue sensation works as a biological alarm and urges us to take a rest. It is essential to adequately rest based on the subjective level of fatigue (i.e., fatigue sensation) to prevent disrupting homeostasis and to recover from fatigue. Therefore, to understand the mechanisms of fatigue, it is important to clarify the neural mechanisms of estimating the subjective level of fatigue that lead us to take a rest.

In our previous study, we focused on the neural mechanisms related to the decision to rest in the presence of fatigue^10^. In this study, while performing reverse Stroop test trials, participants were asked to decide whether to rest or not in order to maintain task performance, and neural activity related to making the decision to rest was recorded using magnetoencephalography (MEG). A decrease of 4–8 Hz band power in the left Brodmann’s area (BA) 31 (i.e., the posterior cingulate cortex [PCC]), 8–13 Hz band power in the left BA 10 and BA 9, and 13–25 Hz band power in the right BA 46 and left BA 10 were observed in relation to making the decision to rest. In addition, a decrease of 4–8 Hz band power in the PCC was positively associated with the subjective level of fatigue experienced while performing reverse Stroop test trials. Because it has been reported that the neural mechanisms related to self-evaluation of the subjective level of physical and mental fatigue involve the PCC^11^,^12^, it can be interpreted that the frontal brain regions, such as BAs 9, 10, and 46, are involved in the decision to rest, and this decision is based on the subjective level of fatigue assessed in the PCC. These are important neural mechanisms related to the decision to rest based on the subjective level of fatigue experienced at the present moment. However, to prevent disrupting homeostasis and concurrently accomplish the assigned workload, it may also be important to control the level of workload based on the estimation of the subjective level of fatigue that will be experienced in the near future. For example, if the subjective level of fatigue in the near future is overestimated, the workload can be reduced, leading to a decline in performance. On the other hand, if the subjective level of fatigue in the near future is underestimated, excessive workload makes it difficult to maintain performance for a long time, also leading to a decline in performance and/or even disrupting homeostasis.

In the present study, we aimed to clarify the neural mechanisms related to predicting the subjective level of fatigue that will be experienced in the future as a first step in examining the neural mechanisms of the decision to rest based on the future level of fatigue. Because the neural mechanisms related to the prediction of the subjective level of fatigue may involve several brain regions, and the temporal sequences among these brain regions may provide valuable clues, we used MEG with high temporal and spatial resolutions to record neural activities related to the prediction of the subjective level of fatigue. We enrolled healthy participants in this study and assessed oscillatory brain activities, which reflect time-locked cortical activities^13^,^14^,^15^ related to the prediction of the subjective level of fatigue in the future. Because it can be hypothesized that the prediction of the subjective level of fatigue includes the process of evaluating the subjective level of fatigue experienced at the time of the prediction, we added a control condition in which self-evaluation of the subjective level of fatigue at the present moment was performed and compared these activities to extract neural activities characteristic to the future prediction.

## Methods

### Participants

Sixteen healthy male volunteers (21.9 ± 1.7 years of age [mean ± SD]) participated in this study. All participants were right-handed according to the Edinburgh Handedness Inventory^16^. Current smokers, individuals with a history of mental or brain disorder, and individuals taking chronic medications that affect the central nervous system were excluded. The Ethics Committee of Osaka City University approved the study protocol (approval number, 3148) and this study was conducted in accordance with the Declaration of Helsinki and the Japanese Ethical Guidelines for Medical and Health Research Involving Human Subjects. All participants provided written informed consent for participation in this study.

### Experimental design

**Figure 1:**
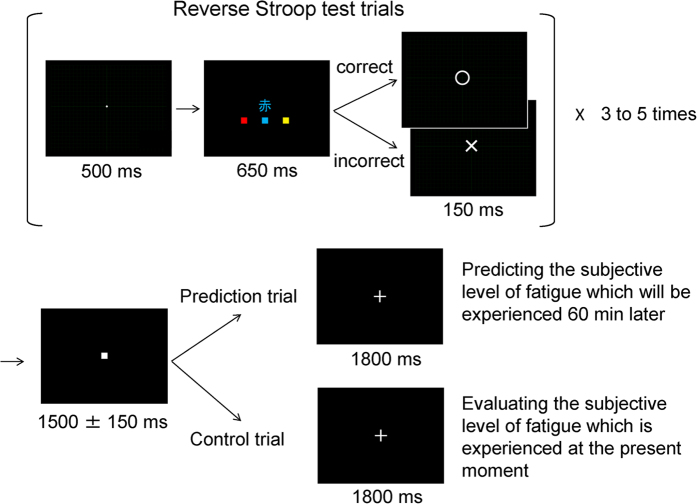
The reverse Stroop test, prediction, and control trials. For the reverse Stroop test trials, a fixation dot was presented on the screen for 500 ms, followed by one of three Japanese characters corresponding to ‘red’, ‘blue’, and ‘yellow’. The character was presented in red, blue, or yellow text. Participants were asked to indicate the meaning of the character, regardless of the color of the text. Participants were instructed to respond as quickly as possible and had a time limit of 650 ms in which to respond. In the prediction condition, after every 3 to 5 reverse Stroop test trials, participants were asked to predict the subjective level of fatigue that would be experienced 60 min later if they continued performing the reverse Stroop test trials (prediction trials). In the control condition, after every 3 to 5 reverse Stroop test trials, participants were asked to evaluate the subjective level of fatigue that was experienced at the time (control trials). The mean ± SD interval between the reverse Stroop test trials and the prediction and control trials was 1500 ± 150 ms. The prediction condition included 120 prediction trials, and the control condition included 120 evaluation trials. Each condition included 320 reverse Stroop test trials.

The experiment consisted of two conditions: a prediction condition and a control condition. The two conditions were conducted in a crossover fashion and on different days. Both in the prediction and control conditions, participants lay on a bed in a magnetically shielded room in the supine position with a screen in front of their eyes, and MEG was recorded (MEG sessions). During the MEG sessions, they performed reverse Stroop test trials projected onto the screen by a video projector (PG-B10S; SHARP, Osaka, Japan). The reverse Stroop test used in this study was the same as one used in our previous study^10^: A fixation dot was presented on the screen for 500 ms, followed by one of three Japanese characters corresponding to ‘red’, ‘blue’, and ‘yellow’. The character was presented in red, blue, or yellow text (Fig. 1). Participants were asked to indicate the meaning of the character, regardless of the color of the text. Responses were made by pressing the left, middle, or right button of a response device (HHSC-1 × 4-L; Current Designs, Philadelphia, PA, USA) with the index, middle, or annular finger of the right hand, respectively. Participants were instructed to respond as quickly as possible and had a time limit of 650 ms in which to respond. Trials in which a button was not pressed within 650 ms were regarded as incorrectly answered. As soon as the participant pressed a button they were provided with feedback as to whether or not their response was correct. Feedback was presented for 150 ms, and was immediately followed by the fixation dot for the next trial.

In the prediction condition, after every 3 to 5 reverse Stroop test trials, participants were asked to predict the subjective level of fatigue that would be experienced 60 min later if they continued performing the reverse Stroop test trials for another 60 min (prediction trials). They were instructed to self-evaluate the subjective level of fatigue experienced at that moment and to predict the subjective level of fatigue that would be experienced 60 min later based on their current subjective level of fatigue. In the control condition, after every 3 to 5 reverse Stroop test trials, participants were asked to evaluate the subjective level of fatigue that was experienced at the time (control trials). They were instructed to predict and to evaluate the subjective level of fatigue in time with visual cues that lasted 1800 ms in the prediction and control conditions. Just before the MEG sessions, they performed the reverse Stroop test, prediction, and control trials to practice predicting and evaluating the subjective level of fatigue, to the point that they thought they were able to perform the prediction and control trials adequately as instructed. The mean ± SD interval between the reverse Stroop test trials and the prediction and control trials was 1500 ± 150 ms (Fig. 1). The jitter was generated based on a Gaussian distribution. The prediction condition included 120 prediction trials, and the control condition included 120 evaluation trials. Each condition included 320 reverse Stroop test trials. The reverse Stroop test trials and the prediction and control cues were created using OpenSesame software^17^.

On each experimental day, the daily level of fatigue was assessed using the Japanese version of Chalder’s fatigue scale^18^,^19^, and the subjective level of fatigue was assessed just before and after the MEG sessions using a 100-mm visual analogue scale (VAS) ranging from 0 (minimum fatigue) to 100 (maximum fatigue). The Japanese version of Chalder’s fatigue scale consists of 11 questions and the response to each question can be 0 (less than usual), 1 (no more than usual), 2 (more than usual), or 3 (more than usual) over the past several weeks^19^.

### MEG recordings

MEG recordings were performed in the MEG sessions using a 160-channel whole-head type MEG system (MEG vision; Yokogawa Electric Corporation, Tokyo, Japan) with a magnetic field resolution of 4 fT/Hz^1/2^ in the white-noise region. The sensor and reference coils were gradiometers with 15.5-mm diameter and 50-mm baseline, and the two coils were separated by 23 mm. The sampling rate was 1,000 Hz, and data were high-pass filtered at 0.3 Hz.

### MEG analyses

The magnetic noise that originated from outside the magnetically shielded room was eliminated by subtracting the data obtained from reference coils using specialized software (MEG 160; Yokogawa Electric Corporation, Tokyo, Japan). Epochs of the raw MEG data that included the artifacts caused by eye blinks and those originated from outside of the magnetically shielded room which remained after the subtraction of the data obtained from reference coils were visually identified and excluded from the analyses before band pass filtering and averaging. The mean numbers of the trials remaining after the rejection of the epochs with artifacts were 82.6 (maximum, 106; minimum 64) and 89.1 (maximum, 119; minimum, 58) for the prediction and control conditions, respectively. Spatial filtering analysis of the MEG data was performed to identify changes in the oscillatory brain activity that reflected time-locked cortical activities^13^,^14^,^15^. The MEG data were bandpass filtered at 8–13 Hz, 13–25 Hz, and 25–58 Hz by a finite impulse response filtering method using Brain Rhythmic Analysis for MEG software (BRAM; Yokogawa Electric Corporation, Tokyo, Japan) to obtain alpha, beta, and gamma signals, respectively. After the bandpass filtering, each epoch in the prediction and control conditions was averaged separately and the location and intensity of the cortical activities were estimated using BRAM, which uses a narrow-band adaptive spatial filtering algorithm^20^,^21^. Voxel size was set at 5.0 × 5.0 × 5.0 mm. For each frequency band and condition (i.e., the prediction and control conditions), the MEG data from 0 to 1650 ms after the onset of the visual cure was divided into 11 segments for 150 ms time window and the oscillatory power of each segment of the MEG data for 150 ms time window relative to that at baseline (from −500 to 0 ms) was calculated (oscillatory power ratio).

Data were then analyzed using statistical parametric mapping (SPM8, Wellcome Department of Cognitive Neurology, London, UK), implemented in Matlab (Mathworks, Natick, MA, USA). The MEG parameters were transformed into the Montreal Neurological Institute T1-weighed image template^22^ and applied to the MEG data. The anatomically normalized MEG data were filtered with a Gaussian kernel of 20 mm (full-width at half-maximum) in the x, y, and z axes. To enable inferences to be made at a population level, individual data were summarized and incorporated into a random-effect model^23^. The weighted sum of the parameters estimated in the individual analysis was used to create “contrast” images that were used for group analyses^23^. The resulting set of voxel values for each comparison constituted a statistical parametric map (SPM) of the t statistic (SPM{*t*}). The SPM{*t*} was transformed to units of normal distribution (SPM{Z}). The significance of the changes in oscillatory band power caused by the prediction and evaluation and the significance of any difference in those between the prediction and control conditions were assessed using t statistics (one sample t test and paired t test, respectively) on a voxel-by-voxel basis^23^. The threshold for the SPM{*t*} of the one sample t test was set at *P* < 0.0015 (familywise-error corrected for multiple comparisons for voxels), considering multiple comparisons among the frequency bands and the time windows. The threshold for the SPM{*t*} of the paired t test was set at *P* < 0.05 (familywise-error corrected for multiple comparisons for voxels).

### Magnetic resonance (MR) image overlay

Anatomical MR imaging was performed using a Philips Achieva 3.0 TX (Royal Philips Electronics, Eindhoven, The Netherlands) to permit registration of magnetic source locations with their respective anatomical locations. Before MR scanning, five adhesive markers (Medtronic Surgical Navigation Technologies Inc., Broomfield, CO, USA) were attached to the skin of the head: two markers 10 mm in front of the left and right tragus, one marker 35 mm above the nasion, and two markers 40 mm to either side of the marker above the nasion. The MEG data were superimposed on MR images using information obtained from these markers and MEG localization coils.

### Statistical analyses

Values are presented as mean and SD unless otherwise stated. Two-way analysis of variance (ANOVA) with repeated measures was performed to assess the effect of condition (the prediction and control conditions) and time point (just before and after the MEG sessions) on the subjective level of fatigue. A paired t test with Bonferroni correction was used to compare the subjective level of fatigue across different time points in the prediction and control conditions. Relationships between changes in oscillatory band power and the VAS scores and those among changes in oscillatory band power were evaluated using Pearson’s correlation analyses. All *P* values were two-tailed, and values less than 0.05 were considered statistically significant. Statistical analyses were performed using IBM SPSS 21.0 software (IBM, Armonk, NY, USA).

## Results

### VAS scores for fatigue sensation

**Figure 2:**
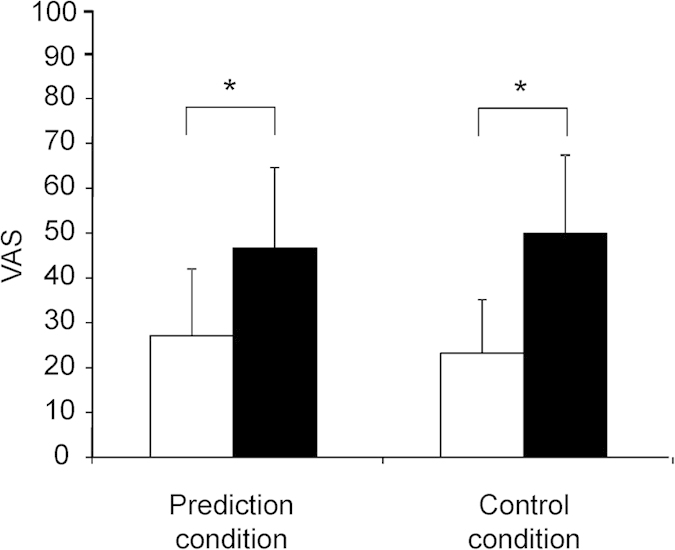
Subjective level of fatigue in the prediction and control conditions. Subjective level of fatigue just before (open column) and after (closed column) the MEG sessions are shown. Participants were asked to rate the subjective level of fatigue on a 100-mm visual analogue scale (VAS) from 0 (minimum fatigue) to 100 (maximum fatigue). Data are presented as mean and SD. *P < 0.01, paired t test with Bonferroni’s correction.

Subjective levels of fatigue assessed by VAS just before and after the MEG sessions in the prediction and control conditions are shown in Fig. 2. Two-way ANOVA with repeated measures was performed to compare the subjective level of fatigue across conditions and time points. There was a main effect of time point [F(1, 15) = 62.205, *P* < 0.001], but there was no main effect of condition [F(1, 15) = 0.011, *P* = 0.918] or condition × time point interaction [F(1, 15) = 3.444, *P* = 0.083]. The subjective level of fatigue just after the MEG session was higher than that just before the MEG session both in the prediction and control conditions (*P* < 0.01, paired t test with Bonferroni correction).

### Daily level of fatigue

The level of the daily level of fatigue in the prediction condition was not different from that in the control condition (*P* = 0.120, paired t test).

### Spatial filtering analyses of MEG data

**Figure 3:**
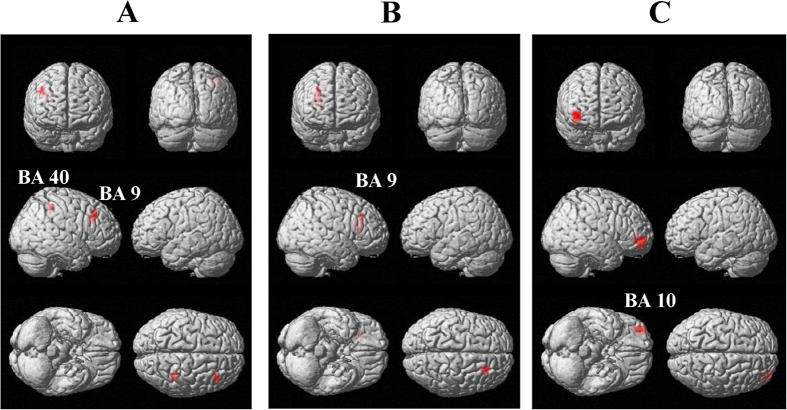
Statistical parametric maps of brain regions where decreases of oscillatory band power were greater in the prediction condition than in the control condition. The decrease of alpha (8–13 Hz) band power in the time window of 1200 to 1350 ms (**A**), that in the time window of 1350 to 1500 ms (**B**), and the decrease of gamma (25–58 Hz) band power in the time window of 1500 to 1650 ms (**C**) are shown. Statistical parametric maps are superimposed on surface-rendered high-resolution MR images. Random-effect analyses of 16 participants, *P* < 0.05, familywise-error corrected for the entire search volumes.

**Figure 4:**
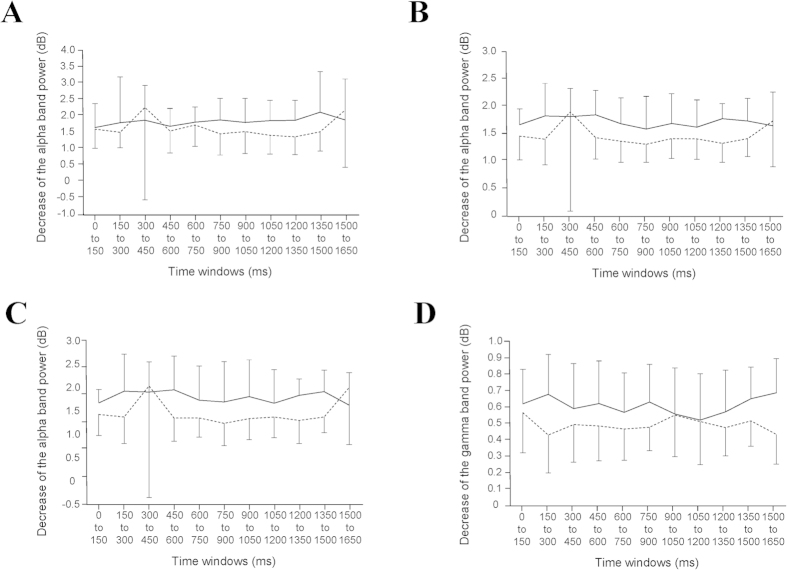
The time course changes of the oscillatory band power at the peak voxels in the brain regions where decreases of oscillatory band power were greater in the prediction condition than in the control condition which are shown in Fig. 3 and Table 1. The time course changes of the alpha (8–13 Hz) band power at the peak voxel in the BAs 40 (**A**) and 9 (**B**) in the time window of 1200 to 1350 ms, those in the BA 9 in the time window of 1350 to 1500 ms (**C**), and those of the gamma (25–58 Hz) band power in the BA 10 in the time window of 1500 to 1650 ms (**D**) are shown. The solid and dotted lines indicate the data in the prediction and control conditions, respectively. The means + SDs and means − SDs are shown for the prediction and control conditions, respectively.

**Figure 5:**
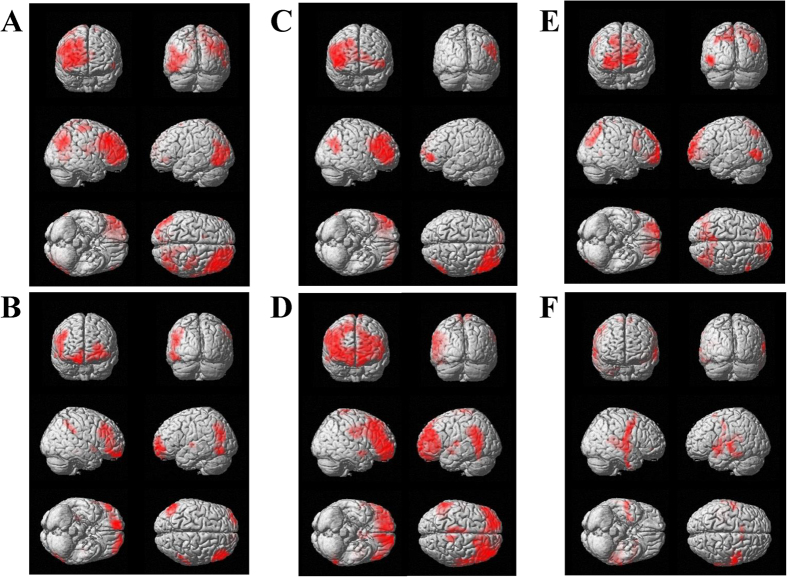
Statistical parametric maps of brain regions where decreases of oscillatory band power were observed in the prediction and control conditions. The decrease in alpha (8–13 Hz) band power observed in the time window of 1200 to 1350 ms in the prediction (**A**) and control (**B**) conditions, that observed in the time window of 1350 to 1500 ms in the prediction (**C**) and control (**D**) conditions, and the decrease in gamma (25–58 Hz) band power in the time window of 1500 to 1650 ms in the prediction (**E**) and control (**F**) conditions are shown. Statistical parametric maps are superimposed on surface-rendered high-resolution MR images. Random-effect analyses of 16 participants, familywise-error corrected for the entire search volumes. For the presentation purpose, the statistical threshold for (**A**–**D**) are set at *P* < 1.0 × 10^−6^ and that for (**E**,**F**) are set at *P* < 1.0 × 10^−5^.

Decreases of the oscillatory power ratio were observed across the frequency bands and the time windows (one sample t test) in the prediction and control conditions. To identify changes in the neural activity caused by predicting subjective level of fatigue that will be experienced in the future, the oscillatory power ratio in the alpha, beta, and gamma frequency bands was compared between the prediction and control conditions. There were several clusters of voxels in which a decrease of oscillatory band power in the prediction condition was significantly greater than that in the control condition (paired t test, Table 1 and Fig. 3). The decrease of alpha band power caused by the prediction in the right BAs 40 and 9 in the time window of 1200 to 1350 ms was significantly greater than that in the control condition (Fig. 3A). The decrease of alpha band power caused by the prediction in the right BA 9 in the time window of 1350 to 1500 was significantly greater than that in the control condition (Fig. 3B). The decrease of gamma band power caused by the prediction in the right BA 10 in the time window of 1500 to 1650 ms was significantly greater than that in the control condition (Fig. 3C). The time course changes of the oscillatory band power at the peak voxel of these four brain regions are shown in Fig. 4. Decrease of beta band power caused by the prediction was not observed in any time windows. For the time windows from 1200 to 1350 ms, 1350 to 1500 ms, and 1500 to 1650 ms, the brain regions in which the decrease of oscillatory band power caused by predicting and evaluating the subjective level of fatigue in the prediction and control conditions, respectively, are shown in Fig. 5 (one sample t test). There were no brain regions in which the increase of the oscillatory power ratio was observed across the frequency bands and the time windows (one sample t test).

### Relationships among the decrease of oscillatory band power in the brain regions that were greater in the prediction condition than in the control condition

**Figure 6:**
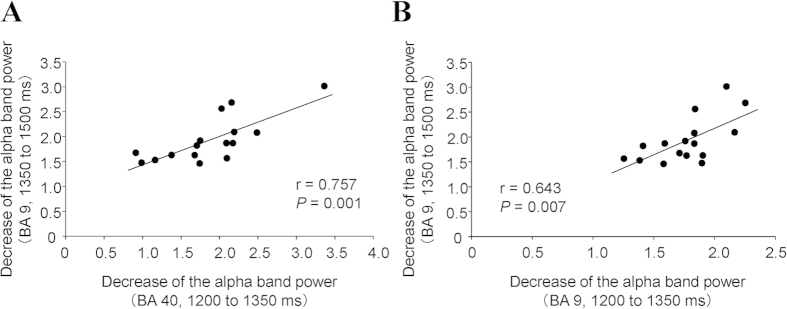
Relationships among decreases of oscillatory band power in the brain regions where findings were greater in the prediction condition than in the control condition. The decrease of alpha band power in the BA 40 in the time window of 1200 to 1350 ms was positively associated with that in the BA 9 in the time window of 1350 to 1500 ms (**A**); *P* = 0.001, r = 0.757). The decrease of alpha band power in the BA 9 in the time window of 1200 to 1350 ms was positively associated with that in the time window of 1350 to 1500 ms (**B**), *P* = 0.007, r = 0.643). Linear regression lines, Pearson’s correlation coefficients, and *P* values are shown.

The decrease of alpha band power in the BA 40 in the time window of 1200 to 1350 ms was positively associated with that in the BA 9 in the time window of 1350 to 1500 ms (*P* = 0.001, r = 0.757; Fig. 6A). The decrease of alpha band power in the BA 9 in the time window of 1200 to 1350 ms was positively associated with that in the time window of 1350 to 1500 ms (*P* = 0.007, r = 0.643; Fig. 6B).

### Relationship between the decrease of oscillatory band power and the daily level of fatigue

**Figure 7:**
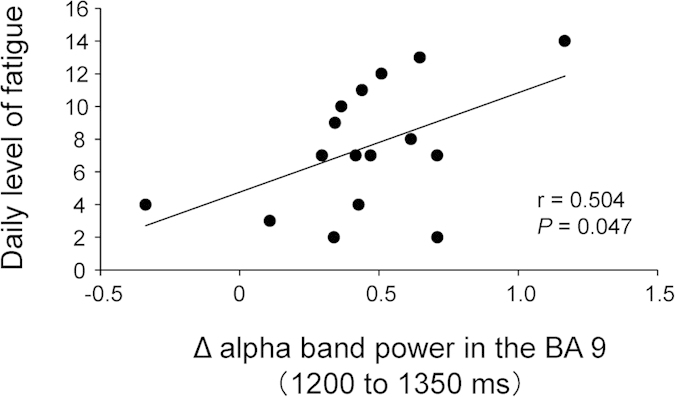
Relationship between decreases of oscillatory band power and daily level of fatigue. For each cluster of voxels in which the decrease in oscillatory band power in the prediction condition was greater than that in the control condition, Δ band power was calculated at the peak voxel of each cluster as follows: Δ band power = oscillatory power ratio in the prediction condition – oscillatory power ratio in the control condition. Δ alpha band power in BA 9 in the time window of 1200 to 1350 ms was positively associated with the daily level of fatigue (*P* = 0.047, r = 0.504). Linear regression line, Pearson’s correlation coefficient, and *P* value are shown.

For each cluster of voxels in which the decrease in oscillatory band power in the prediction condition was greater than that in the control condition, Δ band power was calculated at the peak voxel of each cluster as follows: Δ band power = oscillatory power ratio in the prediction condition – oscillatory power ratio in the control condition. The relationships between the daily level of fatigue and the Δ band power in the BAs 40 and 9 in the time window of 1200 to 1350 ms, that of alpha band power in the BA 9 in the time window of 1350 to 1500 ms, and that of gamma band power in the BA 10 in the time window of 1500 to 1650 ms were assessed. Δ alpha band power in BA 9 in the time window of 1200 to 1350 ms was positively associated with the daily level of fatigue assessed using Chalder’s fatigue scale (*P* = 0.047, r = 0.504; Fig. 7).

## Discussion

In the present study, we examined neural activities related to the prediction of the subjective level of fatigue that will be experienced 60 min later. Decreases of alpha band power in the right BAs 40 and 9 in the time window of 1200 to 1350 ms and that in the right BA 9 in the time window of 1350 to1500 ms, and the decrease of gamma band power in the right BA 10 in the time window of 1500 to 1650 ms were related to the prediction of the subjective level of fatigue. In addition, the decrease of alpha band power in the BA 9 in the time window of 1200 to 1350 ms was positively associated with the daily level of fatigue assessed using Chalder’s fatigue scale, suggesting the involvement of this brain region in the pathophysiology of fatigue.

To the best of our knowledge, this study is the first to report the neural mechanisms of the prediction of subjective level of fatigue in the future. There have been several reports related to the neural mechanisms of thinking of events that will take place in the future. In a functional MRI study in which neural activities related to re-experiencing the past and pre-experiencing the future were investigated, the increases in brain activities in the left BAs 40, 9, and 10 were associated with future event evocation^24^. Another study that used H_2_^15^O PET to examine the neural mechanisms of thinking of the future and past showed that the bilateral BAs 9 and 10 were related to thinking of the future^25^. Our results are consistent with these previous studies in that the BAs 40, 9, and 10 were related to thinking of the future. However, while the laterality of the brain regions was left or bilateral in previous studies, all the brain regions related to the prediction of the subjective level of fatigue were on the right side in the present study. Although the reason for this is unclear from the present study, one possible explanation of this is that the subjective level of fatigue (i.e., fatigue sensation) has emotional aspects: there have been several reports that the processing related to emotions, unpleasant emotions in particular, are primarily processed in the right hemisphere^26^,^27^. The right-sided involvement of our brain regions may be a specific feature of the neural mechanisms of the prediction related to subjective level of fatigue. Furthermore, although it is of great interest to find out the way in which the PCC is involved in the neural mechanisms of predicting subjective level of fatigue, since the process of the self-evaluation of the level of fatigue was included both in the prediction and control conditions in our present study, the role of the PCC in the prediction of the subjective level of fatigue was not determined in the present study.

It is of great interest that the decrease of alpha band power in the BA 9 in the time window of 1200 to 1350 ms was positively associated with the daily level of fatigue. Because it has been reported that the decrease of alpha band power reflects activation of the cortical areas involved in processing of sensory or cognitive information^13^,^28^, the decrease of alpha band power in the BA 9 observed in the present study may reflect the activation of this brain region. It has been reported that the gray-matter volume of the bilateral prefrontal cortex was decreased in patients with chronic fatigue syndrome (CFS), which is an illness characterized by a profound, disabling, and unexplained sensation of fatigue lasting at least 6 months^29^, and the volume reduction in the right BA 9 paralleled the severity of the fatigue of these patients^30^. In patients with chronic pain, it has been reported that the gray matter volume in the prefrontal cortex and thalamus was decreased and it seems that the continuous activation of the brain regions caused by chronic pain induced the release of excitotoxicity and inflammatory agents, resulting in the volume reduction in these brain regions^31^,^32^. Similarly, continuous activation of the BA 9 may occur due to the sustained severe fatigue in patients with CFS and this may cause the neuroinflammation of the BA 9 through the excitotoxicity and inflammatory agents, leading to the volume reduction of the BA 9. Of course, CFS is not thought to be caused by overwork and thus, there is a possibility that the excessive activation of the BA 9 is not the cause of the volume reduction of this brain region in the patients with CFS. In addition, it is difficult to determine cause-and-effect relationships on this point because our present study is a cross-sectional design and included only healthy participants. Therefore, we should be careful to interpret the implication of these findings.

The latency of the changes in oscillatory band power observed in the present study were 1200 to 1350 ms for the BAs 9 and 40 in alpha band power, 1350 to 1500 ms for the BA 9 in alpha band power, and 1500 to 1650 ms for the BA 10 in gamma band power. Because it has been reported that the neural activities related to the self-evaluation of the level of mental fatigue are related to the decrease of delta band power in the dorsolateral prefrontal cortex and the PCC during the time window of 600 to 900 ms ms^12^, the findings that the latency of the changes of oscillatory band power in our present study were more than 900 ms are consistent with the fact that the subjective level of fatigue was predicted after the evaluation of the present level of fatigue. The positive associations of the decrease of alpha band power between the BA 40 in the time window of 1200 to 1350 ms and the BA 9 in the time window of 1350 to 1500 ms, and between the BA 9 in the time window of 1200 to 1350 ms and that of 1350 to 1500 ms indicate that these brain regions work in concert to predict the subjective level of fatigue that will be experienced in the future. Because it has been reported that the frontal pole is one of the brain regions specific to thinking of the future^25^, and the oscillatory brain activity at a higher frequency is reported to reflect involvement of the neurons in smaller brain area^13^, the decrease of gamma band power in the BA 10 observed in the present study may indicate that localized information processing related to the prediction of subjective level of fatigue is performed in the BA 10.

There are limitations to our study. First, the number of participants included in our study was small. To generalize our results, studies with a larger number of participants are needed. Second, participants were asked to predict the subjective level of fatigue that will be experienced only 60 min later. It is of interest whether the distance to the future of which the subjective level of fatigue is predicted affects the neural activity related to the prediction of the subjective level of fatigue. Third, we used reverse Stroop test trials in this study. Neural mechanisms related to the prediction of subjective levels of different types of fatigue, such as physical fatigue, may be different from those caused by reverse Stroop test trials.

In conclusion, we showed that the BAs 40, 9, and 10 are involved in the neural mechanisms of the prediction of the subjective level of fatigue that will be experienced in the future. Impairment or overactivation of these brain regions, in particular the BA 9, seems to be involved in the pathophysiology of chronic fatigue. Our findings may help in the understanding of the neural mechanisms to take a rest based on the prediction of the subjective level of fatigue and the pathophysiology of fatigue-related conditions and/or diseases.

## Additional Information

**How to cite this article**: Ishii, A. *et al*. Neural mechanisms to predict subjective level of fatigue in the future: a magnetoencephalography study. *Sci. Rep.* **6**, 25097; doi: 10.1038/srep25097 (2016).

## Figures and Tables

**Table 1:** Brain regions that showed a greater decrease of oscillatory band power in the prediction condition compared with that in the control condition.

Frequency, latency, and location	Brodmann’s area	MNI coordinates (mm)			Z value
		x	y	z	
8–13 Hz					
1,200 to 1,350 ms					
Right middle frontal gyrus	9	42	28	35	4.07
Right inferior parietal lobule	40	37	−42	50	4.04
1,350 to 1,500 ms					
Right middle frontal gyrus	9	27	28	30	4.12
25–58 Hz					
1,500 to 1,650 ms					
Right middle frontal gyrus	10	37	53	−5	4.08

BA, Brodmann’s area; MNI, Montreal Neurological Institute.

x, y, z: Stereotaxic coordinate.

Data were obtained from random-effect analyses. Only significant changes are shown (paired t test, *P* < 0.05, familywise error rate).
